# Nuclear dynamics: Formation of bodies and trafficking in plant nuclei

**DOI:** 10.3389/fpls.2022.984163

**Published:** 2022-08-23

**Authors:** Eduardo Muñoz-Díaz, Julio Sáez-Vásquez

**Affiliations:** ^1^Centre National de la Recherche Scientifique (CNRS), Laboratoire Génome et Développement des Plantes, UMR 5096, Perpignan, France; ^2^Univ. Perpignan Via Domitia, Laboratoire Génome et Développement des Plantes, UMR 5096, Perpignan, France

**Keywords:** nucleoplasm, nucleolus, stress, localization signals, non-coding RNAs, nuclear boodies

## Abstract

The existence of the nucleus distinguishes prokaryotes and eukaryotes. Apart from containing most of the genetic material, the nucleus possesses several nuclear bodies composed of protein and RNA molecules. The nucleus is separated from the cytoplasm by a double membrane, regulating the trafficking of molecules in- and outwards. Here, we investigate the composition and function of the different plant nuclear bodies and molecular clues involved in nuclear trafficking. The behavior of the nucleolus, Cajal bodies, dicing bodies, nuclear speckles, cyclophilin-containing bodies, photobodies and DNA damage foci is analyzed in response to different abiotic stresses. Furthermore, we research the literature to collect the different protein localization signals that rule nucleocytoplasmic trafficking. These signals include the different types of nuclear localization signals (NLSs) for nuclear import, and the nuclear export signals (NESs) for nuclear export. In contrast to these unidirectional-movement signals, the existence of nucleocytoplasmic shuttling signals (NSSs) allows bidirectional movement through the nuclear envelope. Likewise, nucleolar signals are also described, which mainly include the nucleolar localization signals (NoLSs) controlling nucleolar import. In contrast, few examples of nucleolar export signals, called nucleoplasmic localization signals (NpLSs) or nucleolar export signals (NoESs), have been reported. The existence of consensus sequences for these localization signals led to the generation of prediction tools, allowing the detection of these signals from an amino acid sequence. Additionally, the effect of high temperatures as well as different post-translational modifications in nuclear and nucleolar import and export is discussed.

## Introduction: Cell compartmentalization and the nucleus

Cell compartmentalization allows the physical separation of molecules and metabolic reactions within the cell. In particular, plants possess and exert a large number of biochemical routes and metabolites because of their sessile character (Solymosi and Schoefs, 2019). Thus, compartmentalization is essential in plant cells for their correct functioning. During evolution, compartmentalization appeared as the distinction between eukaryotes and prokaryotes, since prokaryotic cells lack membrane-bound organelles. Interestingly, plant cells possess an exclusive organelle, the chloroplast, whose best-known function consists in obtaining energy through photosynthesis (Alberts et al., 2002; Lunn, 2007; Solymosi and Schoefs, 2019).

**FIGURE 1:**
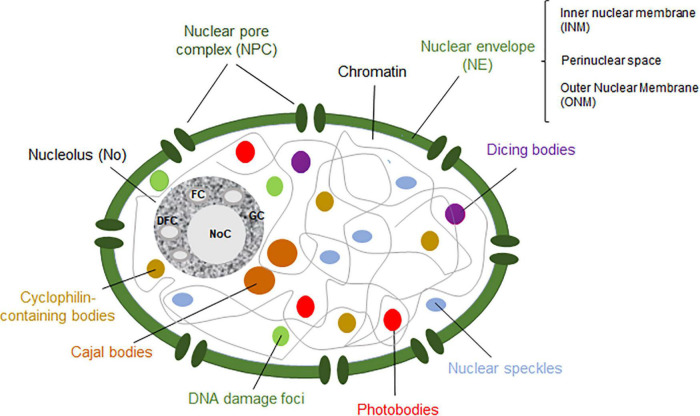
Schematic representation of a plant nucleus showing the nucleolus (dark gray), Cajal bodies (brown), dicing bodies (purple), nuclear speckles (blue), photobodies (red), cyclophilin-containing bodies (light brown) and DNA damage bodies (green). The nuclear enveloppe (NE) and nuclear pore complex (NPC) are shown in green. The fibrillar centers (FCs), dense fibrillar component (DFC), granular component (GC) and nucleolar cavity (NoC) are ilustrated within the nucleolus. Figure based on Petrovská et al. (2015).

Nevertheless, the nucleus can be conceived as the organelle distinguishing eukaryotic and prokaryotic cells. The nucleus contains most of the genetic material, excluding the mitochondrial and (in plants) the chloroplastic genomes (Figure 1). Functionally, it separates the DNA replication and DNA transcription taking place in the nucleoplasm from the protein translation in the cytosol. The nucleus also has a protective effect on the genetic material. Structurally, it comprises the nucleoplasm and the nuclear envelope (NE). The nucleoplasm contains the chromatin and the nuclear bodies, and it is also the site of several enzymes involved in the metabolism of DNA and RNA. On the other hand, the NE delimitates the nucleoplasm from the cytoplasm. It is composed of the outer nuclear membrane and the inner nuclear membrane (ONM and INM, respectively), forming the perinuclear space in-between. Whereas the ONM is in contact with the endoplasmic reticulum in the cytoplasm, the INM associates with the nuclear lamina, which is involved in several nuclear functions in animals cells (Taddei et al., 2004; Guo and Fang, 2014). The nucleoplasm and the cytoplasm are in contact through thousands of nuclear pore complexes (NPCs) located along the NE. In addition, actively transcribed chromatin is often found interacting with the NPC, whereas inactive chromatin is associated with the nuclear lamina in animals and yeast (Taddei et al., 2004; Németh and Längst, 2011) or with the periphery of the nucleolus in eukaryotic cells (Hicks, 2013; Padeken and Heun, 2014; Pontvianne et al., 2016; Picart-Picolo et al., 2020; Castel and Chae, 2021).

Nuclear bodies are dynamic structures composed of proteins and RNA molecules involved in related functions (Mao et al., 2011; Petrovská et al., 2015). They are present in the nucleoplasm and/or the nucleolus. Indeed, the best-known nuclear body in eukaryotic cells is the nucleolus (Mao et al., 2011; Sleeman and Trinkle-Mulcahy, 2014; reviewed by Sáez-Vásquez and Medina, 2008). Even though animals, yeast and plant cells share certain nuclear bodies, there are others that are exclusive to each cell type. On the one hand, nuclear bodies in human cells include the nucleolus, promyelocytic leukemia nuclear bodies, nuclear speckles, paraspeckles, Cajal bodies (CBs), Sam68 bodies, as well as other non-characterized nuclear bodies (Fong et al., 2013). On the other hand, plant nuclear bodies include the nucleolus, CBs, dicing bodies (D-bodies), nuclear speckles, cyclophilin-containing speckles, photobodies and DNA damage foci (Figure 1; Guo and Fang, 2014; Petrovská et al., 2015; Emenecker et al., 2020). Nuclear bodies can be distinguished according to their composition (Table 1) and/or in terms of their assembly mechanism. Three different models have been proposed to describe the formation of nuclear bodies. Firstly, a stochastic series of events may lead to the assembly of nuclear bodies, in which the structural process is mainly random. Moreover, nuclear bodies can also be formed by a coordinated mechanism, in which each element is incorporated into the nuclear body after the other following a tightly sequential order. In this model, only one or two assembly pathways exist to form the nuclear body. Lastly, nuclear bodies can also follow the seedling assembly mechanism, in which one of their components acts as a seed to initiate and nucleate the formation of the nuclear body (Mao et al., 2011; Matera et al., 2011; Guo and Fang, 2014). Whereas the formation of the nucleolus is governed by a seedling mechanism, where the nascent rRNAs act as the “seed” (Karpen et al., 1988), CBs have been observed to follow a stochastic events among the different components (Kaiser et al., 2008).

**TABLE 1:** Plant nuclear bodies.

Nuclear body	Protein components	RNA components	Function
Nucleolus (No)	Nucleolin Fibrillarin …	rRNAs C/D snoRNAs H/ACA snoRNAs	Ribosome biogensis Regulation of the cell cycle Stress response
Cajal bodies (CBs)	Coilin U2B Fibrillarin Dyskerin AGO4, DCL3	snRNAs C/D snoRNAs H/ACA snoRNAs scaRNAs mRNAs	Modification of sRNAs Formation of spliceosomal particles Gene silencing (Arabidopsis)
Dicing bodies (D-bodies)	DCL1 HYL1 AGO1 DRB1 HEN1	-	Gene silencing
Nuclear speckles	SR-rich proteins snRNPs non-snRNPs Transcription factors 3′ processing factors Cyclophilins	snoRNAs pre-mRNAs	Formation of spliceosomal particles
Cyclophilin-containing bodies	Cyclophilins (BypRS64)	-	Protein folding Plant development and signaling
Photobodies	Phytochromes Cryptochromes	-	Storage of active phytochromes Degradation of phytochromes
DNA damage foci	yH2AX RBR1 RAD51, RAD54 E2F	-	DNA damage response (DDR)

The proteins and RNAs listed are considered to be major (abundance) components and normally used as compartmental markers.

In this review, we will consider the different plant nuclear bodies, from their protein and RNA composition to the functions and processes they are involved in. Moreover, the dynamics of nuclear bodies upon different stressors in animals, yeast and plants will be also addressed. In addition, the features of the different protein signals that govern nuclear and nucleolar import and export will be detailed. This review also focuses on how heat stress and main post-translational modifications (PTMs) modulate nuclear import and export.

## Nuclear bodies

### Nucleolus

The nucleolus is the most prominent subnuclear structure in eukaryotic cells. It is considered to be a nuclear body because of the presence of protein and RNA molecules. The nucleolus possesses a tripartite composition distributed in a vectorial fashion: fibrillar center (FC), dense fibrillar component (DFC) and granular component (GC), although in yeast the nucleolus exhibits a bipartite organization (Sáez-Vásquez and Gadal, 2010). The FCs are low-density areas surrounded by the DFC, which is embedded in the GC. Moreover, it is also common to find nucleolar cavities in plant nucleoli (Jordan, 1984; Sirri et al., 2008). Moreover, the plant nucleoli differentiate between two types of FCs: homogeneous (similar to animals) and heterogeneous. Homogeneous FCs, composed of a fibrous loose material, are small and numerous, generally abundant in cells actively producing ribosomes. In contrast, heterogeneous FCs are large and scarce, associated with low translation rates (Sáez-Vásquez and Medina, 2008).

The nucleolus is largely known for its role in the biogenesis of ribosomes (Mélèse and Xue, 1995). Ribosome biogenesis begins with the transcription of rRNA genes (rDNA) into pre-rRNA molecules. The 5.8S, 18S and 25S (28S in mammals) rRNA genes are located in tandem in polycistronic rDNA units (called 35S, 45S, and 47S rDNA in yeast, plants and mammals, respectively) and transcribed by the RNA polymerase I (RNA Pol I). A fourth rRNA gene, the 5S, is transcribed by the RNA Polymerase III (RNA Pol III; Campell et al., 1992). RNA Pol I and III are multimeric enzymatic complexes composed of up to ∼15 subunits (Guilfoyle and Dietrich, 1987; Haag and Pikaard, 2007; Fernández-Tornero et al., 2013; Ream et al., 2015).

Transcribed pre-rRNAs (47S/45S/35S and 5S) undergo several processing steps by endo- and exonucleases to form the mature 5S, 5.8S, 18S and 25S (28S in mammals) rRNAs. In plants, this include endonucleases RTL2 (ribonuclease 3-like protein 2) and 5′-3′ and 3′-5′ exoribonuclease activities from XRN2 and the exosome, respectively (reviewed by Sáez-Vásquez and Delseny, 2019). In the processing of rRNA, both C/D and H/ACA small nucleolar ribonucleoproteins (snoRNPs) also play a central role in the modifications of the rRNAs. On the one hand, C/D snoRNPs are involved in the 2′-*O*-methyl ribose methylation of rRNAs, in which fibrillarin has been described in many species as the methyltransferase. In contrast to animals and yeast, two different genes encode fibrillarin in *Arabidopsis thaliana*, referred as Arabidopsis (AtFIB1 and AtFIB2; Barneche et al., 2000; Pih et al., 2000). Approximately 120 sites experiencing 2′-*O*-methyl ribose methylation have been described in Arabidopsis (Azevedo-Favory et al., 2021). On the other hand, H/ACA snoRNPs mediate 5-riboyluracil pseudouridinyation of rRNAs. In Arabidopsis, dyskerin conforms the catalytic subunit of the H/ACA complex (Maceluch et al., 2001). Dyskerin is encoded by a single gene in Arabidopsis, AtNAP57. In addition, rRNA molecules can be subjected to base methylations, such as m^7^G, m^6^A, m^3^U, m^5^C or Ac^4^C (Sloan et al., 2017; Taoka et al., 2018).

The mature 5S, 5.8S and 25S rRNAs (28S rRNA in mammals), along with large ribosomal proteins (RPLs), form the large ribosomal particle (60S), while the small ribosomal particle (40S) contains the 18S rRNA plus small ribosomal proteins (Fromont-Racine et al., 2003; Korostelev and Noller, 2007; Weis et al., 2015; Sáez-Vásquez and Delseny, 2019).

The number of nucleolar proteins in Arabidopsis is significantly lower than in humans (Palm et al., 2016; Montacié et al., 2017). In Arabidopsis, the vast majority are proteins involved in the transcription and processing of the rRNAs (Leung et al., 2003; Pendle et al., 2005; Montacié et al., 2017). Nucleolin is the most abundant non-ribosomal protein in the nucleolus in eukaryotic cells (Ginisty et al., 1999; Tajrishi et al., 2011; Durut and Sáez-Vásquez, 2015). Even though it is required for ribosome biogenesis, nucleolin participates in other functions such as DNA replication, mRNA stability and translation or maintenance of the chromatin (Roger et al., 2003; Kim et al., 2005; Takagi et al., 2005; Angelov et al., 2006). In Arabidopsis, two different nucleolins are found (AtNUC-L1/NUC1 and AtNUC-L2/NUC2), showing structural homology with animal and yeast nucleolins (Pontvianne et al., 2007; reviewed by Durut and Sáez-Vásquez, 2015).

The assembly and organization of the nucleolus are governed by liquid–liquid phase separation (LLPS; Lafontaine et al., 2021). LLPS consists of the spontaneous demix of a solution into several phases that coexist. Thus, the three nucleolar subdomains (FC, DFC, and GC) behave as three different coexisting liquid phases (reviewed by Emenecker et al., 2020). Many nucleolar proteins, such as fibrillarin or nucleolin, are capable of condensing through LLPS, feature designated as multivalency (reviewed by Banani et al., 2017). These proteins possess Gly-Arg-rich (GAR) domains as well as intrinsically disordered regions (IDRs), which have been observed to promote LLPS. Interestingly, the protein content of the DFC and GC generate immiscibility owing to disfavorable interaction. Thus, the nucleolus can be seen as a multilayered condensate whose formation is governed by LLPS (reviewed by Lafontaine et al., 2021).

During mitosis, the nucleus disappears in the majority of the eukaryotes, including animal and plants. The nucleolus disassembles in the early mitosis, becoming completely lost in the prometaphase. Nevertheless, some nucleolar components appear to be associated with the periphery of the chromosomes during the metaphase and anaphase as sheath-like structures. In the telophase, the sheath-like material forms the perinucleolar bodies (PNBs), which are recruited by the nucleolar organizer regions (NORs). This fact, along with the transcription of the rRNA genes, promotes the synthesis of new nucleoli in the daughter cells (Ochs et al., 1985). Moreover, transcription from Alu elements generates the so-called aluRNAs, which are necessary to maintain the nucleolar integrity (Caudron-Herger et al., 2015). These elements have not yet been described in plants. Exceptionally, many fungi, including yeast, undergo closed mitosis in which the nuclear structures are present throughout mitosis (Asakawa et al., 2016).

### Cajal bodies and histone locus bodies

Cajal bodies are among the best-characterized nuclear bodies in animal, yeast and plant cells. They were discovered by Ramon y Cajal along with other nuclear bodies (reviewed by Nizami et al., 2010). These dynamic structures are able to fuse and divide. Moreover, they are also associated with the nucleolus, moving in or out of it (Andrade et al., 1991; Beven et al., 1995; Boudonck et al., 1999). Whereas the absence of CBs causes developmental abnormalities and lethality in animals (Liu et al., 2009; Walker et al., 2009; Strzelecka et al., 2010; Kanno et al., 2016), CBs are not essential for plant viability (Collier et al., 2006; Nizami et al., 2010). The protein and RNA content of these bodies is very diverse and extensive. The main component of CBs is coilin (Collier et al., 2006), followed by small nuclear RNPs (snRNPs) involved in the processing of pre-mRNAs, such as U2B (Beven et al., 1995); and small nucleolar RNPs (C/D and H/ACA snoRNP) involved in the processing of rRNAs, tRNAs and snRNAs (Ogg and Lamond, 2002; Kannan et al., 2008), as well as signaling pathways (Love et al., 2017). In plants, proteins involved in gene silencing are also part of CBs, such as AGO4 or DCL3 (Li et al., 2006; Pontes and Pikaard, 2008). Coilin is required for the formation of CBs, as shown in knockout and knockdown mutants in some species, i.e., *Arabidopsis thaliana* or *Mus musculus* (Tucker et al., 2001; Collier et al., 2006). The structure of the coilin shows homology across several species: it possesses two nuclear localization signals (NLS), one predicted nucleolar localization signal (NoLS), an N-terminal globular domain and a C-terminal Tudor-like structure (Makarov et al., 2013). Furthermore, three RNA species are localized in CBs: snRNAs, snoRNAs and small CB-specific RNAs (scaRNAs). Interestingly, plant CBs also contain poly(A) RNAs, such as mRNAs (Kim et al., 2010; Niedojadło et al., 2014). Contrary to other types of nuclear bodies, CBs are dynamic because of a continuous exchange of their components (reviewed by Nizami et al., 2010).

Because of the diverse composition of CBs, these nuclear bodies take part in numerous functions. One of the most important processes involving CBs is the formation of spliceosomal particles (snRNPs). After being synthesized in the nucleoplasm, they are translocated to the cytosol to interact with Sm proteins. After methylation of the 5′ of the snRNAs, the snRNP complex moves back into the nucleus (Suzuki et al., 2010). Another function revolving CBs is the modification of small RNAs (sRNAs). The presence of C/D box snoRNAs and scaRNAs mediates the 2′-*O*-methyl ribose methylation of snRNAs, whereas H/ACA box snoRNAs promote 5-riboyluracil pseudouridinyation of RNA molecules in CBs (Bassett, 2012). The possible role of CBs in telomerase activity has been hypothesized. In Arabidopsis, the telomerase interacts with dyskerin, which is a component of CBs. Moreover, the telomerase in invertebrates possesses a domain that leads to accumulation in CBs (reviewed by Love et al., 2017).

Some functions attributed to CBs are specific to plants, i.e., the nonsense-mediated mRNA decay (NMD), a quality control mechanism for premature terminated mRNA molecules. Whereas this process takes place in the cytosol in human cells, the nonsense-mediated mRNA decay might occur in the nucleolus in plants. The nucleolar localization of the exon junction complex, mRNA molecules and Up-frameshift factors in plants sparked the idea of CBs involved in the nonsense-mediated mRNA decay. Nevertheless, this hypothesis must be fully demonstrated (Pendle et al., 2005; Trinkle-Mulcahy, 2009; Bassett, 2012). Another plant-specific function of these bodies is gene silencing. Several components of the gene-silencing machinery have been observed to co-localize with components of CBs (Li et al., 2006, 2008; Fujioka et al., 2007).

Histone locus bodies are another type of nuclear body involved in the processing of histone pre-mRNA, as they are associated with histone-coding genes. In fact, these bodies resemble CBs in terms of structure and composition. HLBs were first discovered in *Drosophila melanogaster* and human cells, even though they were considered to be CBs (Frey and Matera, 1995; Liu et al., 2006; Bongiorno-Borbone et al., 2008; Ghule et al., 2008; Nizami et al., 2010). As CBs, HBLs also contain coilin. The difference resides in the fact that coilin is not essential for the assembly of HLBs, in contrast to CBs (Love et al., 2017). However, these bodies have not been described in plants.

### Dicing bodies

MicroRNAs (miRNAs) are a type of RNase III-dependent sRNAs involved in gene silencing. They are transcribed by the RNA Polymerase II (RNA Pol II) as pri-miRNAs, which are processed by the DCL1-HYL-SE complex into a duplex miRNA. Then, these duplexes associate with ARGONAUTE proteins to form the RISC complex in order to exert their function (reviewed by Liu et al., 2012). In Arabidopsis, DCL1 was found to form round structures in the nucleus. These bodies were able to diffuse around the nucleoplasm, but they were not associated with the nucleolus. In addition, HYL1 also forms aggregates in the nucleus, co-localizing with DCL1 bodies. Similarly, SE forms aggregates in the nucleus. However, they do not always co-localize with DCL1 and HYL, as SE is also found in nuclear speckles (Fang et al., 2004; Fang and Spector, 2007; Song et al., 2007). What is more, the DCL1-HYL1 bodies are different from CBs owing to the absence of coilin. Thus, these DCL1-HYL1 bodies were named D-bodies (Fang and Spector, 2007). Other proteins co-localizing with D-bodies include AGO1, HEN1, DRB1, and PIF4 (reviewed by Emenecker et al., 2020). The formation of D-bodies is also governed by LLPS. It was observed that SE forms droplets, followed by the presence of HYL, DCL1 and pri-/pre-miRNAs. The absence of SE inhibits the formation of D-bodies, which indicates that these bodies are formed via SE-phase separation (Xie et al., 2021).

### Nuclear speckles and paraspeckles

Nuclear speckles constitute another type of common nuclear body present in animal and plant cells (Reddy et al., 2012). These bodies are located in the interchromatin space, and they store splicing factors, as well as snRNPs, non-snRNPs, transcription factors and 3′ processing factors (Lamond and Spector, 2003). These speckles are normally found near active transcription sites, where pre-mRNA molecules have been also found forming fibers (Spector and Lamond, 2011).

Serine/arginine (SR)-rich proteins are splicing proteins involved in recognition of pre-mRNA introns and in the assembly of the spliceosome (Lorkoviæ et al., 2004). The arginine/serine (RS)-rich motif present in these proteins, apart from having an NLS, has been experimentally demonstrated to be responsible for the accumulation in the nuclear speckles (Tillemans et al., 2005). The number, size and shape of nuclear speckles in plant nuclei vary according to the metabolic stage, transcriptional activity or cell type. For instance, actively transcribing cells have numerous small nuclear speckles, whereas inhibition of transcription leads to the formation of larger and less numerous nuclear speckles (Reddy et al., 2012). Interestingly, the co-localization of SR-rich proteins within nuclear speckles also depends on the cell type and/or environmental conditions of the plant cell. What is more, it has been observed that the co-localization of proteins in nuclear speckles does not imply physical interaction among them (Lorkoviæ et al., 2008; Reddy et al., 2012). There is a continuous interchange of components between the nuclear speckles and the nucleoplasm (Rausin et al., 2010). The presence of Arabidopsis SR31, SR1 and atSRp30 in nuclear speckles was demonstrated (Fang et al., 2004). Even though the main components of these bodies are SR proteins, the precise composition of the nuclear speckles continuously changes (Reddy et al., 2012).

Paraspeckles have been described in animals and are composed of non-coding RNA molecules and proteins. They have not been described in plants (Spector and Lamond, 2011; Reddy et al., 2012).

### Cyclophilin-containing bodies

Cyclophilins are a family of proteins that are present in many organelles in plant cells (Singh et al., 2020). They are believed to be involved in protein folding, possibly mediating the assembly of the spliceosome. Recently, it has been observed that cyclophilins constitute versatile proteins that exert a wide array of functions in plant development and signaling (Schmid, 1995; Lorkoviæ et al., 2004; Singh et al., 2020). It was observed that CypRS64, a member of the cyclophilins in Arabidopsis, formed certain bodies in the nucleus named cyclophilin-containing bodies. This protein contains three different domains: (i) the PPiase motif, (ii) the KRS motif, and (iii) the RS/SP domain. The localization of CypRS64 in the cyclophilin-containing bodies requires both the KRS and the RS/SP domains. It is also known that cyclophilins interact with SR proteins, which form part of the nuclear speckles. When CypRS64 was co-expressed with one of its interactors, CypRS64 translocated into the nuclear speckles. It has been hypothesized that this re-localization allows the gathering of different proteins involved in the same process. In addition, the phosphorylation of the CpRS64-interacting proteins is necessary in order to associate with CypRS64 (Lorkoviæ et al., 2004).

### Photobodies

Phytochromes (phys) are photoreceptors responsible for the red (R) and far-red (FR) sensing (Schâfer et al., 1972). They possess inactive and active conformations, referred as R light-absorbing Pr and FR light-absorbing Pfr forms, respectively. In Arabidopsis, there are five types of phys (phyA–phyE). Among them, the most prominent in Arabidopsis are phyA, which senses R, FR and blue light, and phyB, which responds to R light (van Buskirk et al., 2012). Interestingly, the conversion from Pr to Pfr leads to the translocation of phys from the cytosol into the nucleus (Kircher et al., 1999; Kim et al., 2000; Rockwell et al., 2006). Not only are these receptors located in the nucleus upon light excitation, but they also form nuclear bodies, named photobodies (Yamaguchi et al., 1999). The formation of photobodies occurs during the dark-to-light transition. Photobodies containing both phyA and phyB can be observed a few minutes after exposure to R light. These photobodies, named “early photobodies,” disappear after 1 h of exposure. After 2 h of exposure to R light, novel photobodies called “late photobodies” are formed. PhyA is no longer present in the “late photobodies,” as they have been degraded because of continuous exposure to light (Kircher et al., 1999; Yamaguchi et al., 1999; Kim et al., 2000; Bauer et al., 2004). The size and number of phyB photobodies are determined by the amount of phyB in the Pfr form under continuous exposure to R light. Upon high-intensity R light, the Pfr form is predominant, leading to the formation of large photobodies. On the other hand, dim R light generates smaller and more dispersed photobodies, because of the conversion of Pfr into Pr (Chen et al., 2003). In addition, some phyB photobodies may contain cryptochromes, which are blue light receptors (reviewed by van Buskirk et al., 2012). Apart from photoreceptors, the composition of the photobodies includes transcription factors, such as Constans (CO) and the B-box transcription factor 28 (BBX28), or the E3 ligase constitutively photomorphogenic 1 (COP1; Liu et al., 2014, 2020).

Structurally, phys are able to form either homodimers or heterodimers. Each monomer possesses a N-terminal domain to sense light, and a C-terminal domain to allow dimerization (Clack et al., 2009; Nagatani, 2010). The C-terminus of phyB is required for the formation of photobodies independently of light. The proline-rich domain (PRD) domain, in the C-terminus, might contain an NLS or be able to bind a nuclear protein. Notably, the whole C-terminal domain (PRD and histidine kinase-related domain (HKRD) subdomains) is required for the formation of photobodies under normal conditions (Matsushita et al., 2003; Chen et al., 2005). It has been proposed that in the inactive Pr form, the C-terminal domain of phyB is hidden and masked by the N-terminal domain. The transition to the active Pfr form allows the exposure of the NLS in the C-terminal domain to form the photobodies (Fankhauser and Chen, 2008). Regarding the function of photobodies, several hypotheses have been proposed due to the heterogeneous composition: (i) they may act as a storage site for active photoreceptors, (ii) they could be sites of protein degradation, since many proteins are localized in photobodies prior to degradation, or (iii) they could act in transcriptional regulation, as many transcriptional regulators are present (reviewed by van Buskirk et al., 2012). Using a nucleolus-tethering system (NoTS) to dive into the assembly of the photobodies, it was observed that any of the components of the photobodies is able to trigger the formation and assembly of these bodies. Thus, the formation of the photobodies follows a stochastic pathway, also referred as self-organized assembly (Liu et al., 2014).

### DNA damage foci

Because of the sessile character of plants, they are highly exposed to several adverse conditions that lead to DNA damage. However, the mutation rate is very low due to the existence of reparation mechanisms. One of these is called the DNA damage response (DDR), which is highly conserved among animals and plants. The DDR starts with the activation of the protein kinases ataxia telangiectasia mutated (ATM) by double strand breaks (DSBs), and ATM- and Rad3-related (ATR) proteins by single strand (SS) DNA. Then, the suppressor of gamma-response 1 (SOG1) is phosphorylated, promoting the transcription of DNA repair genes and the regulation of the cell cycle. However, there is a SOG1-independent DDR, which involves E2F-retinoblastoma-related protein 1 (RBR1) complexes (reviewed by Nisa et al., 2019).

DNA damage foci appear at sites of double-stranded damage in animals, yeast and plants. It has been hypothesized that LLPS, similar to the nucleolus, governs the formation of these bodies (reviewed by Emenecker et al., 2020). The protein components of DNA damage foci are involved in DDR. One of those proteins is the phosphorylated histone H2AX (γH2AX). This histone variant accumulates at DNA damage sites, becoming an excellent marker for DNA damage foci (Löbrich et al., 2010). In Arabidopsis, the E2F transcription factors also form DNA damage foci that co-localize with γH2AX. In addition, RBR1 co-localizes with E2Fa in DNA damage foci, recruiting proteins involved in DNA repair (Lang et al., 2012; Biedermann et al., 2017). Other components of the DNA damage foci include radiation-sensitive (RAD) proteins, such as RAD54 (Hirakawa and Matsunaga, 2019) or RAD51 (Kurzbauer et al., 2012). However, according to Singh et al., RAD51 did not co-localized with γH2AX. Moreover, the gamma-tubulin complex component 3-interacting protein 1 (GIP1), involved in the maintenance of the nuclear structure and organization, forms nuclear foci that co-localize with γH2AX foci (Singh et al., 2022).

## Nuclear granules and bodies under stress

**FIGURE 2:**
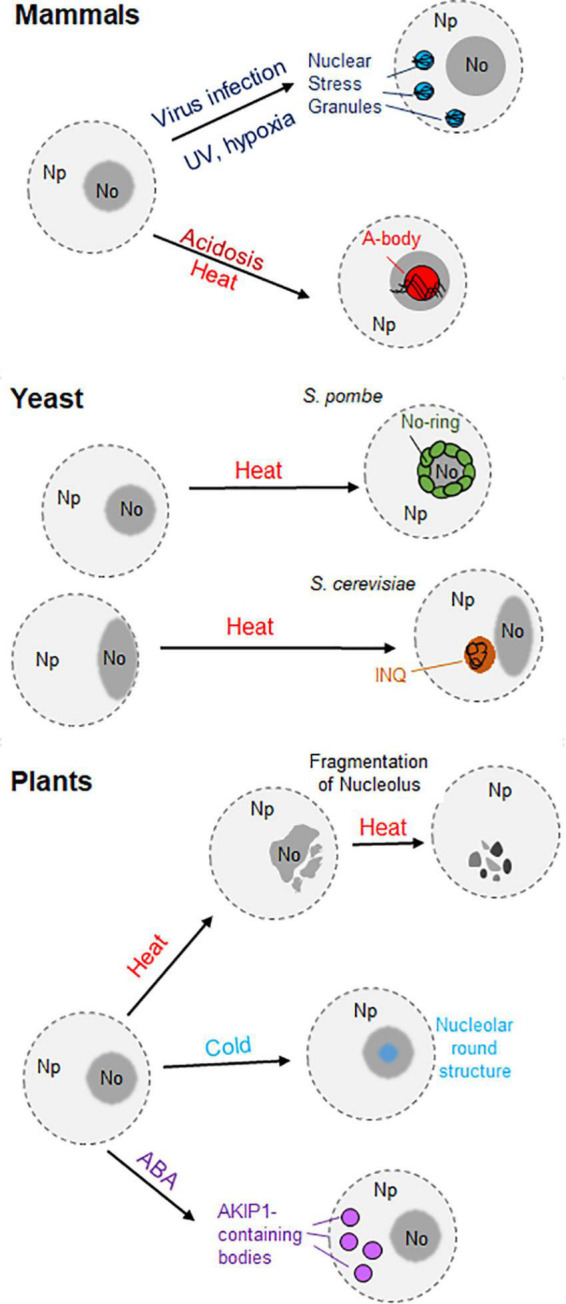
Representation of the different nuclear aggregates originating under stress conditions in mammals, yeast, and plants. In mammal cells (top) two different nuclear granules are represented: the nuclear stress granules (SGs), formed in the nucleoplasm (Np) upon viral infection, hypoxia or UV exposure; and the A-bodies, formed in the nucleolus (No) in response to acidosis or heat stress. In the middle, the generation of nuclear aggregates in yeast is driven by acute heat stress. While nuclear/nucleolar proteins form the nucleolar ring (No-Ring) in fission yeast (*Schizosaccharomyces pombe*), the intra-nuclear quality-control compartment (INQ) appears in the nuclei of budding yeast (*Saccharomyce cerevisiae*) in contact with the nucleolus. On the bottom, the loss of the nucleolar structure occurs in plant cells upon heat stress, whereas the presence of a round structure occurs during chilling temperatures. Moreover, the formation of AKIP1-containing bodies in the nucleus was observed in *Vicia faba* in the presence of abscisic acid (ABA).

Eukaryotic cells are often exposed to unfavorable conditions, such as extreme temperatures or hypoxia. These stresses activate different types of cellular responses to mitigate and/or fight the adverse conditions (Audas et al., 2016). These stressors have been demonstrated to have an impact on the composition, shape, size and number of nuclear bodies. For instance, the nucleolus undergoes reversible changes in response to low and high temperatures. Plant nucleoli show speckled structures upon incubation at 37°C (Hayashi and Matsunaga, 2019). Moreover, they start to disaggregate and dissemble after longer exposure to 37°C (Darriere et al., 2022). On the other hand, chilling temperatures lead to the formation of a round structure in the nucleolus. It was also observed that both low and high temperatures inhibit the accumulation of newly synthesized rRNA in the nucleolus (Figure 2; Hayashi and Matsunaga, 2019).

High temperatures induce the enlargement of CDKC2-containing nuclear speckles, whereas cold treatment inhibits their formation (Kitsios et al., 2008; reviewed by Reddy et al., 2012). CBs also respond to heat shock, since they disappear upon exposure to high temperatures. Nevertheless, they reappear once the heat stress stops (Boudonck et al., 1999). As coilin plays a role in some signaling pathways in plant cells, the involvement of CBs and/or coilin has been suggested in the perception and response to stresses (Love et al., 2017).

Different cellular bodies are formed in response to abiotic stress. In the cytosol of plant cells, stress granules and heat stress granules appear upon short- and long-term exposure to heat stress, respectively. They can also be differentiated according to their protein and RNA composition (reviewed by Maruri-López et al., 2021). Interestingly, the generation of nuclear aggregates upon diverse stimuli was observed in human cells and yeast (Figure 2). The nuclear stress granules appear in the nucleoplasm of mammalian cells after exposure to different stimuli, such as hypoxia or UV exposure (Figure 2). However, their existence in plant cells remains uncharacterized (Gaete-Argel et al., 2021; reviewed by Biamonti and Vourc’h, 2010).

Moreover, the amyloid bodies (A-bodies) are formed in the nucleoplasm of human cells in response to various stimuli such as hypoxia, heat stress or acidosis (Figure 2). The protein content of these A-bodies [also referred as Detention Center in Audas et al. (2012a)], is heterogeneous, but all of the proteins share a protein motif known as an amyloid-converting motif (ACM). In addition, the presence of lncRNAs derived from the ribosomal intergenic spacer (IGS) was observed in the A-bodies (Audas et al., 2016). Regarding fungi, the formation of nucleolar rings is attributed to *S. pombe*. Upon acute heat stress, nuclear and nucleolar proteins accumulate in the periphery of the nucleolus (Gallardo et al., 2020). Another example constitutes the “intra-nuclear quality-control compartment” located in the nucleus of *S. cerevisiae* upon heat stress. This nuclear structure, located close to the nucleolus, contains misfolded cytosolic and nuclear proteins (Figure 2; Kaganovich et al., 2008; reviewed by Gallardo et al., 2021).

In Arabidopsis, abiotic stress modulates the composition of certain nuclear bodies. For instance, early flowering 3 (ELF3), a component of the evening complex, forms nuclear speckles in response to high temperatures. The C-terminal prion domain of ELF3 is responsible for this behavior (Jung et al., 2020). In contrast, low and high temperatures promote the disaggregation of phyB photobodies in Arabidopsis Col-0 and *Ler* ecotypes. This disaggregation occurs because of the transition from the active Pfr form to the inactive Pf form of phyB photobodies (Legris et al., 2016; Hahm et al., 2020). On the other hand, the recruitment of the RNA-binding proteins UBA2a and UBA2b to nuclear speckles in Arabidopsis is enhanced upon exposure to abscisic acid, a hormone that mediates the response to some abiotic stresses, such as salinity or drought (Bove et al., 2008). Similarly, the RNA-binding protein AKIP1 forms a plant-specific nuclear body called “AKIP1-containing bodies” in fava bean (*Vicia faba*) upon exposure to abscisic acid (Figure 2; Li et al., 2002).

## Moving into and out of the nucleus

**FIGURE 3:**
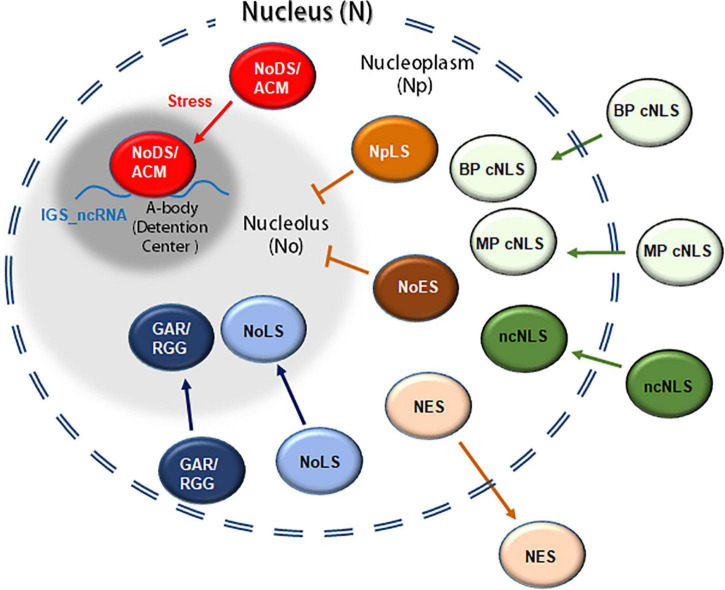
Representation of proteins containing nuclear and/or nucleolar signals involved in their translocation between the cytoplasm, the nucleoplasm and/or the nucleolus. A classical nuclear localization signal (cNLS) including monopartite and bipartite classical signals (MP and BP cNLS, respectively); ncNLS, non-classical nuclear localization signals; NES, nuclear export signal; NoLS, nucleolar localization signal; GAR, glycine arginine rich domain. In mammalian cells certain proteins might also contain an nucleoplasmic localization signal (NpLS), nucleolar exclusion signals (NoES) and/or amyloid-converting motif (ACM, also referred as nucleolar detention signal or NoDS). In response to heat/acidosis the ACM interacts with IGS-derived lncRNA, forming the A-bodies (or detention centers).

In order to exert their function, nuclear proteins, synthesized in the cytosol, need to cross the NE through the NPC. As mentioned before, the NPCs are embedded in the nuclear membrane, creating a channel between the cytoplasm and the nucleoplasm. These cylindrical structures constitute the largest macromolecular complexes present in eukaryotic cells. Morphologically, each NPC is composed by a cytoplasmic and a nucleoplasmic ring, connected by eight spokes. A basket-like structure has been observed in the nucleoplasmic side of the NPC, whereas some fibrillar structures are present in the cytoplasmic face. The main component of the NPC are proteins known as nucleoporins, which are partially conserved in eukaryotes (Nigg, 1997; Stewart, 2007; Hicks, 2013; Petrovská et al., 2015). The NPC regulates protein movement from the cytosol into the nucleus and vice versa. Small molecules can cross the NPC by diffusion. In contrast, larger molecules such as proteins need to be actively translocated in order to cross the NPC (Hicks, 2013). Generally, the exclusion size of the nucleus ranges from 30 to 60 kDa, in which passive transport through the NPC is possible. However, smaller proteins have been observed to cross the NPC by active transport (Timney et al., 2016). Particular amino acid sequences and/or arrangements have been implicated in the active transport of proteins between the cell nucleus and the cytoplasm (Figure 3).

### Nuclear import

The nuclear import mechanism includes the movement of proteins from the cytosol into the nucleus. The molecular players in this mechanism have been characterized in eukaryotes. In animals, there are different nuclear import pathways depending on the protein–protein interactions (Stewart, 2007).

The classical nuclear import pathway includes the cytosolic importin β. This protein interacts with the target nuclear protein through an adaptor protein, importin α, which recognizes a specific motif of the target nuclear protein. The complex importin α–nuclear protein–importin β migrates into the nucleoplasm via interaction with proteins from the NPC. In the nucleus, Ran-GTP promotes the dissociation of importin α, importin β and the target nuclear protein. Importin α moves back into the cytoplasm by interaction with the β-karyopherin CAS and Ran-GTP. At the same time, importin β, along with Ran-GTP, migrates back to the cytoplasm. The hydrolysis of Ran-GTP into Ran-GDP by Ran GTPase-activating protein allows its dissociation from importin β (Smith et al., 1998; Sazer and Dasso, 2000; Hicks, 2013). Finally, the nuclear transport factor 2 (NTF2) mediates the re-importing of Ran-GDP into the nucleus (Kutay and Bischoff, 1997; Ribbeck et al., 1998). In plants, homologs of the components of the machinery have been identified. For instance, Arabidopsis possesses orthologs of importin α, such as At-IMP α and AtKAP α, and impotin β (Ballas and Citovsky, 1997; Hübner et al., 1999; Tamura and Hara-Nishimura, 2014). Moreover, orthologs of importin α and importin β have also been found in rice (Matsuki et al., 1998). This suggests that the nuclear import pathway is mostly conserved between animals and plants.

The specific sequence of the target nuclear protein recognized by importin α is known as the nuclear localization signal (NLS). Unlike other localization signals, such as mitochondrial and plastid signals, NLSs are not proteolytically removed after nuclear import, allowing nuclear proteins to participate in more than one round of nuclear transport. These localization signals can be found in the N- and/or C-terminus, as well as within the protein (Martoglio and Dobberstein, 1998; Lu et al., 2021). Adam et al. described the first NLS in the simian virus 40 large T-antigen (SV40), comprising seven hydrophobic residues (^126^PKKKRKV^132^). Sequence analysis of other nuclear proteins revealed the presence of NLSs. These signals can be classified in different groups according to their structure and composition. The first class includes the classical NLSs (cNLSs), which are the most characterized. This class can also be subdivided into two categories: monopartite and bipartite cNLSs. Monopartite cNLSs are composed of 4–8 basic amino acids, at least four of them being positively charged (lysine or arginine). The consensus sequence for this subgroup of cNLSs is K-K/R-X-K/R, where X represents any residue (Dingwall and Laskey, 1991; Lange et al., 2010; Lu et al., 2021). One of the most notorious and best-characterized members of the monopartite cNLSs subgroup is SV40. Functional analysis of this NLS revealed that the third lysine (^126^PKKKRKV^132^) is necessary for the correct nuclear localization of the protein (Kalderon et al., 1984). The second subgroup within the cNLSs is the bipartite cNLSs, having two clusters of positively charged amino acid residues separated by a spacer of 9–12 residues. The consensus sequence of this subgroup is R/K-X_(9–12)_-K-R-X-K, where X represents any amino acid. The protein nucleoplasmin exemplifies the possession of a bipartite cNLS (^155^KRPAATKKAGQAKKKK^170^, where the two positively charged clusters are underlined; Smith et al., 1995; Lange et al., 2010; Lu et al., 2021). Lange et al. demonstrated the importance of the length of the spacer in the bipartite cNLSs, it being crucial in the interaction with importin α. By testing different spacer lengths of a bipartite cNLS, they concluded that the longer the spacer is, the less nuclear accumulation is observed.

The second class of NLSs is known as non-classical NLSs (ncNLSs). This type comprises NLSs whose composition varies from positively charged residues. The best-known ncNLSs are the proline-tyrosine (PY) ncNLSs, which are composed of 20–30 residues with a basic or hydrophobic N-terminus and a common C-terminal motif ([basic/hydrophobic]-X_*n*_-R/H/K-X_(2–5)_-P-Y, where X represents any amino acid; Wang et al., 2012; Mallet and Bachand, 2013). Nevertheless, other ncNLSs cannot be represented as a consensus sequence, such as the ribosomal protein L23a (Jäkel and Görlich, 1998). Additionally, Lu et al. designated a third class of miscellaneous NLSs, including (i) proteins with a potential NLS, predicted *in silico*, that do not lead to nuclear localization; (ii) NLSs recognized upon protein dimerization; (iii) cryptic NLSs, where a stimulus is necessary for the translocation into the nucleus; and (iv) proteins with multiple NLSs, all of them required for nuclear import.

In plants, several NLSs have been characterized both *in silico* and experimentally. Examples include the E3 ubiquitin-protein ligase COP1 in Arabidopsis, a repressor of the photomorphogenesis. This protein exhibits nuclear localization owing to the presence of a bipartite cNLS (^294^RKKRIHAQFNDLQECYLQKRRQLA^317^; Stacey and Von Arnim, 1999). Another example is the Arabidopsis transcriptional elongation regulator MINIYO, which possesses two NLSs in its sequence. One of them is a monopartite cNLS located in the N-terminus (^254^LKKRKH^259^), whereas the other is a bipartite cNLS present in the C-terminus (^1401^RKRHREGMMLDLLRYKK^1417^; Contreras et al., 2019). Arabidopsis RTL2 contains a bipartite cNLS in the C-terminal portion (^371^KKAESSSAYHMIRALRK^387^; Comella et al., 2008). In maize, three different NLSs are present in the protein R. Two of them are monopartite cNLSs (^100^CDRRAAPARP^109^ located in the N-terminus, and ^419^MSERKRREKL^428^ found within the sequence). The third NLS is a Mat α2-type NLS, named after the unusual NLS of the Mat α2 protein in yeast (Hall et al., 1984), located in the C-terminus (^598^ MISESLRKAICKR^610^; Shieh et al., 1993; Hicks et al., 1995). Finally, the first 43 amino acids of the *Brassica napus* 60S ribosomal protein L13-1 are sufficient to target this protein to the nucleus. The NLS is likely to be present between the residues 29 and 43 (^29^RKTRRRVARQKKAVK^43^; Table 2; Sáez-Vásquez et al., 2000).

**TABLE 2:** Nuclear localization signals.

Protein	Organism	Sequence^1^	Type	References
**Nuclear localization signals (NLS)**				
COP1	*Arabidopsis thaliana*	^294^RKKRIHAQFNDLQECYLQKRRQLA^317^	BP cNLS	Stacey and Von Arnim (1999)
MINIYO	*Arabidopsis thaliana*	^254^LKKRKH^259^	MP cNLS	Contreras et al. (2019)
		^1401^RKRHREGMMLDLLRYKK^1417^	BP cNLS	
RTL2	*Arabidopsis thaliana*	^371^KKAESSSAYHMIRALRK^387^	BP cNLS	Comella et al. (2008)
Hsfa1	*Arabidopsis thaliana*	^230^KEKKSLFGLDVGRKRR^245^	BP cNLS	Evrard et al. (2013)
Coilin	*Arabidopsis thaliana*	^175^KRKK^178^	MP cNLS	Makarov et al. (2013)
		^264^KKAKR^268^	MP cNLS	
Protein R	*Zea mays*	^100^CDRRAAPARP^109^	MP cNLS	Hicks et al. (1995)
		^419^MSERKRREKL^428^	MP cNLS	
		^598^ MISESLRKAICKR^610^	ncNLS	
60S ribosomal protein L13-1	*Brassica napus*	^29^RKTRRRVARQKKAVK^43^	N/S	Sáez-Vásquez et al. (2000)
Nucleoplasmin	*Xenopus laevis*	^155^KRPAATKKAGQAKKKK^170^	BP cNLS	Dingwall et al. (1988)
ERK5	*Homo sapiens*	^505^RKPVTAQERQREREEKRRRRQERA KEREKRRQERE^539^	BP cNLS	Kondoh et al. (2006)
CCTα	*Homo sapiens*	^12^RKRRK^16^	MP cNLS	Taneva et al. (2012)
SV40	Simian virus	^126^PKKKRKV^132^	MP cNLS	Adam et al. (1989)
N protein	Porcine reproductive and respiratory syndrome virus	^10^KRRK^13^ ^7^	MP cNLS	Rowland and Yoo (2003)
		^41^PGKKNKK^4^	MP cNLS	

cNLS, classical nuclear localization signal; ncNLS, non-classical nuclear localization signal; MP, monopartite; BP, bipartite; N/S, non-specified. ^1^The two positively charged amino acid clusters of the BP cNLSs are underlined.

### Nuclear export

In contrast to nuclear import, the mechanisms that govern the movement of proteins from the nucleoplasm to the cytoplasm have been much less characterized. There are few examples of transporters of proteins from the nucleoplasm into the cytoplasm. One of them is Exportin1 (CRM1), which is known to specifically interact with nuclear proteins in order to translocate them into the cytoplasm (Fornerod and Ohno, 1997; Ossareh-Nazari et al., 1997). For this, CRM1 interacts with a specific sequence of the nuclear protein, as well as with Ran-GTP. This complex interacts with the NPC, crossing the NE and reaching the cytosol. The complex dissociates when Ran-GTP is hydrolyzed into Ran-GDP (Bischoff and Görlich, 1997; Neville et al., 1997). This nuclear transporter has also been found in Arabidopsis, named AtXPO1. Similarly, AtXPO1 interacts with a specific motif of the nuclear proteins and with the protein Ran in order to export the nuclear proteins (Haasen et al., 1999). It has been suggested that the nuclear proteins react with phenylalanine and glycine (FG)-repeats of the nucleoporins from the NPC in order to reach the cytoplasm (Stutz et al., 1996).

The specific region of the nuclear proteins that is recognized by CRM1/AtXPO1 is called the nuclear export signal (NES). The existence of NESs was initially described in the HIV-1 Rev protein (^75^LPPLERLTLD^84^), involved in the export of pre-mRNAs and mRNAs from the nucleus, and the heat-stable inhibitor (PKI) of cAPK (^38^LALKLAGLDI^47^; Fischer et al., 1995; Wen et al., 1995). These NESs are rather short and hydrophobic sequences where Leu is highly present (Nigg, 1997). The presence of NESs is mainly characterized in proteins involved in the export of RNA molecules from the nucleus. For instance, the translocation of the 5S rRNA to the cytosol is mediated by TFIIIA in Xenopus oocytes, which contains a Rev-like NES (^357^SLVLDKLTI^365^; Guddat et al., 1990). In Arabidopsis, a Rev-like NES is located in the C-terminus of RanBP1a (^171^DTAGLLEKLTVEETKTEEKT^190^; Haasen et al., 1999). Besides a bipartite cNLS, Arabidopsis RTL2 also possesses an NES in the N-terminus (^7^PEYNFPAITRCSLSNSLPHR^26^). The presence of an NLS and NES allows RTL2 to move between the cytosol and the nucleus (Table 3; Comella et al., 2008). The characterization of later NESs allowed grouping the majority of the NESs into three different consensus sequences: φ-X_1,2_-[∧P]- φ -[∧P]_2,3_- φ -[∧P]- φ (class 1), φ -[∧P]- φ -[∧P]_2_- φ -[∧P]- φ (class 2), and φ -X-[∧P]- φ -[∧P]_3_- φ -[∧P]_2_- φ (class 3), where φ represents large hydrophobic residues, X_1,2_ represents any one or two amino acids, [∧P] represents any amino acid except proline, and [∧P]_2,3_ represents any two or three amino acids except proline (Kosugi et al., 2014).

**TABLE 3:** Nuclear export signals and nucleocytoplasmic shuttling signals.

Protein	Organism	Sequence	References
**Nuclear export signals (NES)**			
RanBP1a	*Arabidopsis thaliana*	^171^DTAGLLEKLTVEETKTEEKT^190^	Haasen et al. (1999)
RTL2	*Arabidopsis thaliana*	^7^PEYNFPAITRCSLSNSLPHR^26^	Comella et al. (2008)
TFIIIA	*Xenopus laevis*	^357^SLVLDKLTI^365^	Guddat et al. (1990)
PKI	*Homo sapiens*	^38^LALKLAGLDI^47^	Wen et al. (1995)
N protein	Porcine reproductive and respiratory syndrome virus	^106^LPTHHTVRLIRV^117^	Rowland and Yoo (2003)
Rev protein	HIV-1	^75^LPPLERLTLD^84^	Fischer et al. (1995)
**Nucleocytoplasmic Shuttling Signals (NSS)**			
hnRNP A1	*Homo sapiens*	^316^GNYNNQSSNFGPMKGGNFGGRSSGPYGGGGQYFAKPRNQGGY^357^	Siomi and Dreyfuss (1995)
DAZAP1	*Homo sapiens*	^383^GPPAGGSGFGRGQNHNVQGFHPYRR^407^	Lin and Yen (2006)
RNA helicase A	*Homo sapiens*	^1151^GSTRYGDGPRPPKMARYDNGSGYRRGGSSYSGGGYGGGYSSGG YGSGGYGGSANSFRAGYGAGVGGGYRGVSRGGFRGNSGGDYRGPS GGYRGSGGFQRGGGRGAYGTGY^1260^	Tang et al. (1999)

### Bidirectional signals

Another type of **localization** signal that mediates nucleocytoplasmic trafficking has been described. In contrast to the NLSs or NESs, which exert a unilateral translocation of proteins, the nucleocytoplasmic shuttling signals (NSSs) allow both import and export of proteins to/from the nucleus in human cells. Many NSS-containing proteins interact with mRNA molecules. These motifs are longer and lack basic residues (Michael, 2000). The first NSS was found in the human heterogeneous nuclear ribonucleoprotein A1 (hnRNP A1), which associates with pre-mRNA and mRNA molecules. The domain responsible for the bidirectional behavior of A1 is called M9, present in the C-terminus (see Table 3 for sequence; Siomi and Dreyfuss, 1995). Other examples include the C-terminal ZNS domain of the DAZ-associated protein 1 (DAZAP1; see Table 3 for sequence; Lin and Yen, 2006) or the C-terminal nuclear transport domain (NTD) of the human RNA helicase A (see Table 3 for sequence; Tang et al., 1999). M9-dependent nuclear import is conferred by transportin. Transportin binds directly to the M9 domain, binds to nucleoporins and is sufficient for the movement of M9-containing cargo through the NPC and into the nucleus. This transport seems to be dependent on the Ran-GTP concentration (reviewed by Michael, 2000). To our knowledge, NSSs have not been described in plant proteins.

## Moving into and out of the nucleolus

### Nucleolar localization signal

Once in the nucleus, proteins can diffuse in the nucleoplasm or migrate into the nucleolus and/or other bodies. Unlike nuclear targeting, the mechanism and localization signals that regulate the translocation of proteins into the nucleolus remain highly unexplored (Figure 3). Over the years, several proteins have been predicted to possess a nucleolar localization signal (NoLS). These signals are rich in basic amino acids, especially Lys and Arg, preferentially located in the C- or N-terminus of proteins. Moreover, the NoLSs are predicted to be present in alpha helices or random coils located on the surface of the protein (Scott et al., 2010). For a peptide to achieve nucleolar localization it must be positively charged, formed exclusively of six or more arginines, and with an isoelectric point above 12.6 (Martin et al., 2015).

The existence of NoLSs have been predicted *in silico* and experimentally confirmed. For instance, both isoforms of the Arabidopsis ribosomal protein RPL23a (RPL23aA and RPL23aB) accumulate in the nucleolus owing to an NLS/NoLS region (Degenhardt and Bonham-Smith, 2008). An NoLS was also found in the sequence of Arabidopsis coilin (^202^KKKKKKK^208^), as well as two NLSs (^175^KRKK^178^ and ^264^KKAKR^268^; Makarov et al., 2013). Likewise, the human coilin also presents one NoLS (^160^KKNKRNL^168^), which was experimentally confirmed to be necessary for nucleolar localization (Hebert and Matera, 2000). Moreover, the sequence of the breast cancer autoantigen nucleolar GTP-binding protein 2 (NGP-1) contains two NoLSs. One of them is located in the N-terminus (see Table 4 for sequence), whereas the second one is a C-terminal NoLS (see Table 4 for sequence; Datta et al., 2015). Interestingly, the nucleocapsid (N) protein of the porcine reproductive and respiratory syndrome virus (PRRSV) exhibits both cytosolic and nucleolar localization. This dual accumulation is achieved by the presence of two NLSs (^10^KRRK^13^ and ^41^PGKKNKK^47^), one NoLS (^41^PGKKNKKKNPEKPHFPLATEDDVRHHFTPSER^72^) and one NES (^106^LPTHHTVRLIRV^117^; Table 4; Rowland and Yoo, 2003).

**TABLE 4:** Nucleolar localization signals and nucleolar exclusion signals.

Protein	Organism	Sequence	References
** Nucleolar Localization Signals (NoLS)**			
Coilin	*Arabidopsis thaliana*	^202^KKKKKKK^208^	Makarov et al. (2013)
RPL23a^1^	*Arabidopsis thaliana*	^33^KKDK^36^	Degenhardt and Bonham-Smith (2008)
		^36^KKIR^39^	
Coilin	*Homo sapiens*	^160^KKNKRNL^168^	Hebert and Matera (2000)
NGP-1^2^	*Homo sapiens*	^1^MVKPKYKGRSTINPSKASTNPDRVQGAGGQNMRDRATIRRLNM YRQKERRNSRGKIIKPLQYQSTVASGTVARVEPNIKWFGNTRVIKQ SSLQKFQEEMD^100^	Datta et al. (2015)
		^631^DEKIAKYQKFLDKAKAKKFSAVRISKGLSEKIFAKPEEQRKTLEED VDDRAPSKKGKKRKAQREEEQEHSNKAPRALTSKERRRAVRQQRP KKVGVRYYETHNVKNRNRNKKKTNDSEGQ KHKRKKFRQKQ^701^	
N protein	Porcine reproductive and respiratory syndrome virus	^41^PGKKNKKKNPEKPHFPLATEDDVRHHFTPSER^72^	Rowland and Yoo (2003)
** Nucleolar Export Signals (NoES)**			
GNL3L^3^	*Homo sapiens*	^292^EVYLDKFIRLLDAPGIVPGPNSEVGTILRNCVHVQKLADPVTPVET ILQRCNLEEISNYYGVSGFQTTEHFLTAVAHRLGKKKKGGLYSQEQAAK AVLADWVSGKISFYIPPPATHTLPTHLSAEIVKEMTEVFDIEDTEQAN EDTMECLATGESDELLGDTDPLEMEIKLLHSPMTKIADAIENKTTVYKI GDLTGYCTNPNRHQMGWAKRNVDHRPKSNSMVDVCSVDRRSVLQRI METDP^531^	Meng et al. (2007)
NGP-1^3^	*Homo sapiens*	^349^QYITLMRRIFLIDCPGVVYPSEDSETDIVLKGVVQVEKIKSPEDHIG AVLERAKPEYISKTYKIDSWENAEDFLEKLAFRTGKLLKGGEPDLQTV GKMVLNDWQRGRIPFFVKPPNAEPLVAPQLLPSSSLEVVPEAAQNNP GEEVTETAGEGSESIIKEETEENSHCDANTEMQQILTRVRQNFGKINV VPQFSGDDLVPVEVSDLEEELESFSDEEEEEQEQQRDDAEESSSEPEEE NVGNDTKAVIKAL DEKIAKYQKFLDKAKAKKFS^620^	Meng et al. (2007)
TdIF2/ERBP	*Homo sapiens*	^441^VLLVL^445^	Fukada et al. (2019)

^1^These sequences are described as NLS/NoLS.

^2^The N- and C-terminal regions are responsible for the nucleolar accumulation; the sequence of the NoLS is not detailed.

^3^These sequences are named nucleoplasmic localization signals (NpLSs).

It is widely thought that the nucleolar localization of many proteins is the result of the association with nucleolar components, such as rRNA (Schmidt-Zachmann and Nigg, 1993; Scott et al., 2010). In mammal cells, many ribosomal components adopt nucleolar localization by interaction with B23, the major constituent of the granular component of the nucleolus (Borer et al., 1989). It was proposed that B23, because of its constant movement between the nucleolus and the cytosol, is able to shuttle NoLS-containing proteins into the nucleolus (Borer et al., 1989; Scott et al., 2010). Nevertheless, this hypothesis has not been confirmed. What has been observed is that the ADP-ribosylation factor GTPase-activating protein 1 (ARF GAP 1) accumulates in the nucleolus because of the interaction with B23 (Korgaonkar et al., 2005; Sirri et al., 2008). Interestingly, human nucleolin has not been demonstrated to possess an NoLS, but it does contain a bipartite cNLS (^256^KRKKEMANKSAPEAKKKK^273^). The structure of the nucleolin was analyzed to determine which domain is responsible for its nucleolar accumulation (Créancier et al., 1993; Schmidt-Zachmann and Nigg, 1993). It was proposed that the GAR domain located in its C-terminus is necessary for its localization in the nucleolus (Pellar and DiMario, 2003). The localization of a GAR-deleted nucleolin is mainly nuclear, decreasing its nucleolar accumulation. Nevertheless, there are other domains that are also necessary for the translocation of nucleolin into the nucleolus, such as the RNA recognition motif (RRM). Thus, it was proposed that it interacts with nucleolar components such as rRNA to accumulate in the nucleolus (Doron-Mandel et al., 2021; Okuwaki et al., 2021). Similarly, the N-terminal GAR domain present in the human fibrillarin was demonstrated to drive both nuclear and nucleolar accumulation (Snaar et al., 2000; Shubina et al., 2020). Whereas fibrillarin is located in the nucleolus, GAR-deleted fibrillarin is distributed in the nucleolus, nucleoplasm and cytoplasm. Moreover, the methylation of the arginine residues of the GAR domain positively regulates nuclear localization. However, this methylation decreases the nucleolar accumulation of fibrillarin (Shubina et al., 2020). As previously stated, two nucleolin (AtNUC-L1/NUC1 and AtNUC-L2/NUC2) and fibrillarin (AtFIB1 and AtFIB2) protein genes were described in Arabidopsis. Both nucleolin and fibrillarin proteins contain GAR domains in the C-terminus or N-terminus sequences, respectively. In contrast, the N-terminal region of AtNUC-L1/NUC1 contains two potential bipartite NLSs, whereas there is only one NLS in AtNUC-L2/NUC2 (Barneche et al., 2000; Pontvianne et al., 2007).

### Nucleoplasmic localization signal

Two different types of signals that prevent nucleolar accumulation have been characterized in humans (Meng et al., 2007; Fukada et al., 2019). On the one hand, Meng et al. coined the term nucleoplasmic localization signal (NpLS) to describe the regions of the guanine nucleotide-binding protein-like 3-like protein (GNL3L) and the nucleolar GTP-binding protein 2 (NGP-1) that prevented localization in the nucleolus and promoted nucleoplasmic accumulation. Fukada et al. also identified a region in the terminal sequence of the deoxynucleotidyltransferase-interacting factor 2/estrogen receptor α-binding protein (TdIF2/ERBP) that led to the similar nucleoplasmic localization, called the nucleolar exclusion signal (NoES). Whereas the described NpLSs represent rather large regions (>200 residues), the NoES is only composed of five hydrophobic residues. Each of the NpLSs contains five hydrophobic residues, which indicates that the NpLS and NoES are structurally similar (Table 4; Fukada et al., 2019).

In terms of composition, NoLSs and NLSs have a similar composition, since both contain basic residues. In some cases, nucleolar signals were initially predicted to be nuclear signals because of their similarity. Thus, the experimental validation of predicted nucleolar signals, as well as nuclear signals, is fundamental to fully characterizing the nuclear and/or nucleolar localization of a protein. A classification of NLSs and NoLSs consists of (i) NLS-only signals, responsible for nuclear localization, (ii) NoLS-only signals, which determine localization exclusively in the nucleolus, and (iii) joint NLS–NoLS regions, which lead to accumulation of proteins in the nucleus and the nucleolus (Scott et al., 2010). Likewise, leucine is a common residue of NESs and NoESs. Nevertheless, the NoES characterized by Fukada et al. (^441^VLLVL^445^) cannot be included in any of the consensus NESs described above.

## Nuclear and nucleolar accumulation under heat stress: Amyloid-converting motif/nucleolar detention signal

Part of the heat stress response includes inhibition or induction of specific protein activities. For that, transcription factors (TFs) are responsible of transforming the perception of the stressor into the expression of key genes. More specifically, heat stress transcription factors (HSFs) play a central role in gene transcription under different abiotic stresses, including heat stress (Guo et al., 2016). Consequently, the nuclear proteome undergoes substantial changes upon high temperatures, promoting the accumulation of HSFs. For instance, Arabidopsis bZIP18 and bZIP52, which are present in the cytoplasm under normal conditions, accumulate in the nucleus under heat stress. This nuclear localization is provoked by the dephosphorylation of Ser residues (Wiese et al., 2021). In contrast, phosphorylation of Arabidopsis HsfA2 promotes nuclear accumulation under heat stress. The phosphorylated residue consists of threonine (Thr249) located close to a bipartite cNLS (^230^**KEKK**SLFGLDVG**RKRR**LTST^249^, where the NLS is in bold, and Thr249 is underlined; Evrard et al., 2013). Another example includes the Arabidopsis heat shock factor-binding protein (AtHSBP), which shuttles from the cytosol to the nucleus in response to heat stress (Hsu et al., 2010). Nevertheless, HSFs are not the only proteins that accumulate into the nucleus upon heat stress. Arabidopsis heat–intolerance 4 (HIT4), involved in the release from transcriptional gene silencing, translocates from the chromocentres to the nucleolus under heat stress (Wang et al., 2015).

This nuclear translocation upon heat stress has also been described in human cells. As mentioned previously (see the section: Nuclear granules and bodies under stress), some proteins are retained in the nucleus, forming A-bodies in response to heat stress. This phenomenon was initially called nucleolar sequestration of proteins, including the heat shock protein 70 (Hsp70) or the E3 ubiquitin-protein ligase MDM2. The ACM, which is the peptide responsible for this behavior, was originally defined as a nucleolar detention signal (NoDS). These signals are characterized by the presence of an arginine motif (R-R-L/I) and two or more hydrophobic triplets (L-φ-L/V, where φ represents a hydrophobic residue). Likewise, there is a physical interaction between the NoDS and IGS-derived lncRNAs, transcribed in response to high temperatures (Mekhail et al., 2007; Audas et al., 2012a,b). These NoDS/ACM signals have not been functionally characterized in plants.

## *In silico* prediction tools for nuclear and nucleolar signals

The existence of consensus sequences of localization signals, such as NLSs or NESs, allows their prediction using computational methods. There is a wide array of online platforms and *in silico* methods to predict the existence of NLSs from an amino acid sequence. For instance, “NLStradamus” is based on a Hidden Markov Model and used to find cNLSs from yeast sequences (Nguyen Ba et al., 2009). Cokol et al. (2000) created “predictNLS” by performing *in silico* mutagenesis of a library of 91 experimentally tested NLSs. Similarly, “NESmapper” allows the detection of NESs with high accuracy and a low false positive rate. To do that, every residue of the NES is considered, contributing independently and additively to the nuclear exportation (Kosugi et al., 2014). In the case of “NLSdb,” the server allows the prediction of NLSs and NESs from nine different species, including *Arabidopsis thaliana*, *Homo sapiens* and *Oriza sativa* (Bernhofer et al., 2018).

On the other hand, the prediction of nucleolar signals is challenging. First of all, the only well-stablished nucleolar signal is the NoLS, since few nuclear export signals have been described (see the above NoESs and NpLSs). Moreover, NoLSs are considerably similar to NLSs, both containing arginine and lysine residues. The web server “NoD” appears to be the best tool to predict an NoLS. It predicts the presence of an NoLS from the protein sequence, using the human-trained artificial neural network. Even though “NoD” performs best using mammal and mammalian-infecting viral proteins, it can also be used with plant and plant virus proteins (Scott et al., 2010, 2011).

## Post-translational modifications and nuclear localization

Post-translational modifications consist of the addition of functional groups, or even cleavage, of certain domains. These modifications change the properties of proteins, promoting functional diversity. The best-known PTMs conform the addition of chemical groups, which can be reversible or irreversible, including phosphorylation, methylation, acetylation, or ubiquitination. These modifications have an impact on many aspects of the protein, i.e., activity, half-life, interaction with other molecules, or subcellular localization (Ramazi and Zahiri, 2021). More specifically, certain PTMs are able to modulate the accumulation of certain proteins in the nucleus.

### Phosphorylation

Phosphorylation is the best-characterized PTM. The addition of a phosphate negatively or positively influences the function of a protein. To couple and uncouple phosphate groups, kinases and phosphatases are necessary, respectively. Phosphorylation can also enhance or inhibit nuclear transport through different mechanisms (Nardozzi et al., 2010). The first case portrays the accumulation in the nucleus after protein phosphorylation. As exemplified by Nardozzi et al., phosphorylation can occur in the NLS or in the surrounding sequence. Phosphorylation can also induce conformational changes in the NLS-containing protein, exposing the NLS and enhancing nuclear accumulation. For instance, phosphorylation upstream the cNLS of SV40 (^110^PSSDDEAAADSQHAAPP**KKKRKV**G^133^, where the NLS is marked in bold, and the phosphorylation sites are underlined) enhances nuclear import (Xiao et al., 1997). On the other hand, phosphorylation of proteins lacking NLSs can also increase nuclear accumulation. Upon phosphorylation of the TEY domain of ERK1/2 (^232^LDQLNHILGILGSPSQEDL^250^, the nuclear transport signal sequence, where the phosphorylated residue is underlined) or the RS domain of ASF/SF2, these proteins shuttle from the cytosol into the nucleus (reviewed by Nardozzi et al., 2010). What is more, the phosphorylation of the TEY domain of ERK5 promotes nuclear accumulation. This protein contains a bipartite cNLS and non-classical NES, in which phosphorylation benefits the nuclear import over cytoplasmic localization (Table 2; Kondoh et al., 2006; Nardozzi et al., 2010).

Several examples in which phosphorylation enhances nuclear import in plants can be found in the literature. For instance, the Arabidopsis ssDNA binding protein WHIRLY (WHY1) is present in the nucleus and in the chloroplast under normal conditions. When WHY1 is phosphorylated by the calcineurin B-like-interacting protein kinase 14 (CIPK14), it accumulates predominantly in the nucleus (Ren et al., 2017). Another example is the movement of 14-3-3 proteins from the cytosol to the nucleus upon phosphorylation by the cold-activated plasma membrane protein cold-responsive protein kinase 1 (CRPK1; Liu et al., 2017). The second scenario is the inhibition of the nuclear import upon phosphorylation. Nardozzi et al., presented different cases and examples. On the one hand, phosphorylation of nucleoporins of the NPC can repress nuclear import (Porter and Palmenberg, 2009). On the other hand, the phosphorylation of the NLS provokes cytoplasmic accumulation. As mentioned above, Arabidopsis bZIP18 and bZIP52, which are present in the cytoplasm under normal conditions, accumulate in the nucleus under heat stress. This nuclear localization is provoked by the dephosphorylation of Ser residues (Wiese et al., 2021). In contrast, phosphorylation of Arabidopsis HsfA2 promotes nuclear accumulation under heat stress. The phosphorylated residue consists of threonine (Thr249) located close to a bipartite cNLS (^230^**KEKK**SLFGLDVG**RKRR**LTST^249^, where the NLS is in bold, and Thr249 is underlined; Evrard et al., 2013).

In lymphocyte T cells, the nuclear factor of activated T cells (NFAT) has an NLS and NES. Low Ca^2+^ levels cause phosphorylation of the SRR2 region, which overlaps with the NLS, promoting cytoplasmic localization of the NFAT. On the other hand, nuclear accumulation of the NFAT has been observed with high levels of Ca^2+^ owing to dephosphorylation (Kehlenbach et al., 1998; Belfield et al., 2006). In Arabidopsis, the phytochrome-interacting factor 7 (PIF7) accumulates in the cytosol under white-light conditions because of its phosphorylation. In contrast, shade exposure activates phosphatases, de-phosphorylating PIF7 and promoting its accumulation in the nuclear photobodies (Huang et al., 2018). Similarly, the activity of the transcription factor Brassinazole-resistant 1 (BZR1) is linked to its phosphorylation status. Brassinosteroid-insensitive-2- (BIN2-) mediated phosphorylation of BZR1 promotes nuclear export of BZR1 and cytosolic accumulation. Either de-phosphorylation or mutation of the putative phosphorylation sites in BZR1 results in nuclear accumulation (Ryu et al., 2007).

### Acetylation and methylation

The addition of an acetyl group is catalyzed by acetyltransferases using acetyl CoA as a cofactor. In contrast, the removal of the acetyl group is catalyzed by deacetylases. Protein acetylation becomes necessary in certain situations, such as protein–protein interaction, chromatin stability or nuclear transport (Yang and Seto, 2008; Choudhary et al., 2009; Xia et al., 2020). Some examples in the literature exemplify the role of acetylation in nuclear protein accumulation. For instance, the human tyrosyl-tRNA synthetase (TyrRS) becomes highly acetylated upon oxidative stress, promoting its nuclear accumulation (Cao et al., 2017). Similarly, the acetylation of the Lys10 of the human translational corepressor CtBP2 by the nuclear acetylase p300 is essential for its nuclear localization (Zhao et al., 2006). In contrast, the acetylation of the NLS of some proteins prevents nuclear accumulation. This is the case of the human tyrosine-protein kinase c-Abl, in which the acetylation of the Lys730 within its second NLS (^728^SSKRFLR^734^, in which the Lys730 is underlined) promotes cytosolic localization rather than nuclear import (di Bari et al., 2006).

From another perspective, the acetylation of a component of the nuclear import machinery has an impact on the actual nuclear import rate in Arabidopsis. For instance, acetylation of the Lys18 of Nup50, which promotes the dissociation of importin α from the nuclear protein, decelerates this dissociation, repressing the nuclear import (Matsuura and Stewart, 2005; Füßl et al., 2018).

Protein methylation is a reversible PTM that occurs in the nucleus. Even though many residues can be methylated, this PTM is most common in arginine and lysine (reviewed by Ramazi and Zahiri, 2021). The effects of methylation in nuclear accumulation are diverse and specific for each protein. On the one hand, the human Hsp70 localizes in the nucleus when the Lys561 is dimethylated, accumulating in the cytosol when unmethylated (Cho et al., 2012). On the other hand, the methylation of the Lys494 of Yes-associated protein (Yap) by a SET-domain-containing lysine methyltransferase prevents its nuclear import, remaining in the cytosol in mouse (Oudhoff et al., 2013).

### SUMOylation and Ubiquitination

The attachment of a small ubiquitin-like modifier (SUMO) to proteins involves three different enzymes: (i) the activating enzyme or E1, (ii) the conjugating enzyme or E2, and (iii) the ligase or E3. This PTM is crucial in several processes, such as the regulation of the cell cycle, subcellular localization or transcription (reviewed by Hannoun et al., 2010). In the case of the human polo-like kinase 1 (PLK1), the SUMOylation of the Lys492, which is close to one of the NLSs, is essential for the nuclear accumulation of PLK1, as well as increasing its stability (Wen et al., 2017). Similarly, the SUMOylation of the Lys248 of the human X-linked zinc finger transcription factor ZIC3 is important for its nuclear retention (Chen et al., 2013). As with acetylation, the SUMOylation of the Lys909 of the yeast importin Kap114 is essential for its role in the nuclear import mechanism (Rothenbusch et al., 2012). In Arabidopsis, heat stress increases the amount of SUMOylated proteins in the nucleus, suggesting that SUMOylation induces nuclear import (Saracco et al., 2007).

Ubiquitination is a reversible PTM in which the C-terminus of an active ubiquitin is attached to a protein. Even though ubiquitination can occur in all 20 amino acids, it is more frequent in lysine residues. Similar to SUMOylation, the ubiquitin junction requires three enzymes: (i) the activating enzyme or E1, (ii) the conjugating enzyme or E2, and (iii) the ligase or E3. This reversible modification is normally associated with protein degradation via ubiquitin-proteasome. However, some effects in nuclear translocation have been described (Lecker et al., 2006; Bhogaraju and Dikic, 2016; Ramazi and Zahiri, 2021). The ubiquitination of the Lys57 of the human cytidylyltransferase (CCTα) promotes cytosolic accumulation. This ubiquitination event occurs near the N-terminal NLS of CCTα, disrupting its interaction with importin α (Trotman et al., 2007). Similarly, the monoubiquitination of two lysine residues (Lys13 and Lys289) in PTEN is necessary for its correct nuclear import, where it exerts its role as tumor suppressor (Trotman et al., 2007). Moreover, ubiquitination is also attributed to nuclear export. Upon proteasome inhibition, ubiquitinated proteins accumulate in the cytosol. This accumulation results from the transport of the ubiquitinated proteins from the nucleus to the cytosol (Hirayama et al., 2018). On the other hand, how ubiquitination affects nuclear and/or nucleolar import and/or export in plants remains mainly uncharacterized.

## Perspectives

The purpose of this review is to gather global information concerning nuclear bodies in plants and, in particular, to survey their composition (proteins and RNA) and the peptide or amino acid sequence/structure signals involved in their localization and assembly. We did not intend to present an exhaustive catalog of protein and molecular bases involved in the assembly of nuclear bodies in plants, but rather to establish the current state of the art of these bodies in response to environmental conditions in plants and, more specifically, in response to abiotic stresses. Comparison of the behaviors of conserved nucleolar bodies revealed certain functional and structural similarities in yeast, animal and plant cells. However, under specific environmental conditions, particular nuclear bodies are formed and/or reorganized distinctly. For instance, this pertains to the nucleolus under heat stress conditions. Thus, although under optimal growth conditions nuclear bodies might have similar functions, key differences might appear upon specific developmental and environmental conditions. This is particularly true for plants, which are sessile organisms subjected to major developmental programs (including seed germination and flowering) and constrained to adapt to or resist stressful conditions (biotic and abiotic) to survive. The functional, structural and molecular clues of these bodies remain elusive and deserve further study to better understand the underlying molecular mechanism of nuclear bodies in plants.

## Author contributions

EM-D and JS-V wrote the review. Both authors contributed to the article and approved the submitted version.
